# West Nile virus - a re-emerging global threat: recent advances in vaccines and drug discovery

**DOI:** 10.3389/fcimb.2025.1568031

**Published:** 2025-05-15

**Authors:** Deren Zehra Kocabiyik, Lizdany Flórez Álvarez, Edison Luiz Durigon, Carsten Wrenger

**Affiliations:** ^1^ Department of Parasitology, Institute of Biomedical Sciences, University of São Paulo, São Paulo, Brazil; ^2^ Institut Pasteur de São Paulo, São Paulo, Brazil; ^3^ Department of Microbiology, Institute of Biomedical Sciences, University of São Paulo, São Paulo, Brazil

**Keywords:** West Nile virus, antiviral drug discovery, structural and non-structural protein targets, envelope protein, flavivirus, arbovirus, NS5, NS3

## Abstract

West Nile virus (WNV) is an emerging mosquito-borne pathogen and is posing significant global health challenge through climate change. WNV, transmitted between birds and *Culex* mosquitoes, has significantly expanded northward in recent years, leading to outbreaks across Europe and North America. This review explores the recent advancements and therapeutic strategies targeting WNV’s structural and non-structural (NS) proteins, which play critical roles in viral replication and pathogenesis. Promising candidates include peptide-based inhibitors, monoclonal antibodies, and small molecules that disrupt protein-protein interactions. Most of current findings are derived from *in silico* methods or *in vitro* assays, with limited validation through *in vivo* studies. Although no vaccines are currently available for humans, several have been approved for horses, and development efforts are ongoing. The growing threat of WNV underscores the urgent need for validated antiviral therapies and scalable vaccines, especially considering its increasing geographic range and public health impact.

## Introduction

West Nile virus (WNV) is an emerging mosquito-borne pathogen and a global health challenge, affecting millions of people worldwide through geographical expansion of mosquito vectors due to climate change (Weaver and Reisen, 2010; Weaver, 2013; Erazo et al., 2024). The virus follows an enzootic transmission cycle, spreading between migratory birds and Culex mosquitoes and infecting humans and mammals like horses, which are dead-end hosts (Weaver and Reisen, 2010). Discovered in Uganda in 1937 (Lanciotti et al., 2002), it reached the US by 1999 (Lanciotti et al., 1999), and by 2003, became a leading arthropod-borne disease (DeFelice et al., 2017).

Climate change, particularly rising temperatures and increased rainfall, has fuelled WNV’s spread across Europe, with annual outbreaks in Mediterranean and central regions since the 1990s, particularly in Romania, Italy, and Greece (Paz, 2015; Vogels et al., 2017; Bakonyi and Haussig, 2020; Semenza et al., 2022; Farooq et al., 2023; Giesen et al., 2023; Erazo et al., 2024). Northern regions, including Spain, the Netherlands, Germany, and Hungary, have also been affected, with the largest European outbreak recorded in 2022, causing 1,340 locally acquired cases and 104 deaths (Bakonyi and Haussig, 2020; Farooq et al., 2023; ECDC, 2024a; ECDC, 2024b). WNV spread from North America to Latin America, yet large human outbreaks have not been reported in the latter regions, likely due to underreporting, misdiagnosis, cross-protection from other flaviviruses, or the circulation of attenuated viral strains. In Asia, WNV neuroinvasive cases have been reported in Pakistan, Sri Lanka, and India, but little is known about its impact in other countries. Strengthening surveillance and diagnostic efforts are crucial to prevent potential future epidemics (May et al., 2011; Kramer et al., 2019; Chowdhury and Khan, 2021; Lorenz and Chiaravalloti-Neto, 2022). While most infections are asymptomatic, less than 1% lead to neuroinvasive diseases such as encephalitis or meningitis, particularly in elderly or immunocompromised individuals (Sinigaglia et al., 2020). With no specific vaccine or antiviral treatment available, the increasing incidence of severe cases underscores the urgent need for drug discovery (European Centre for Disease Prevention and Control, 2024).

**Figure 1:**
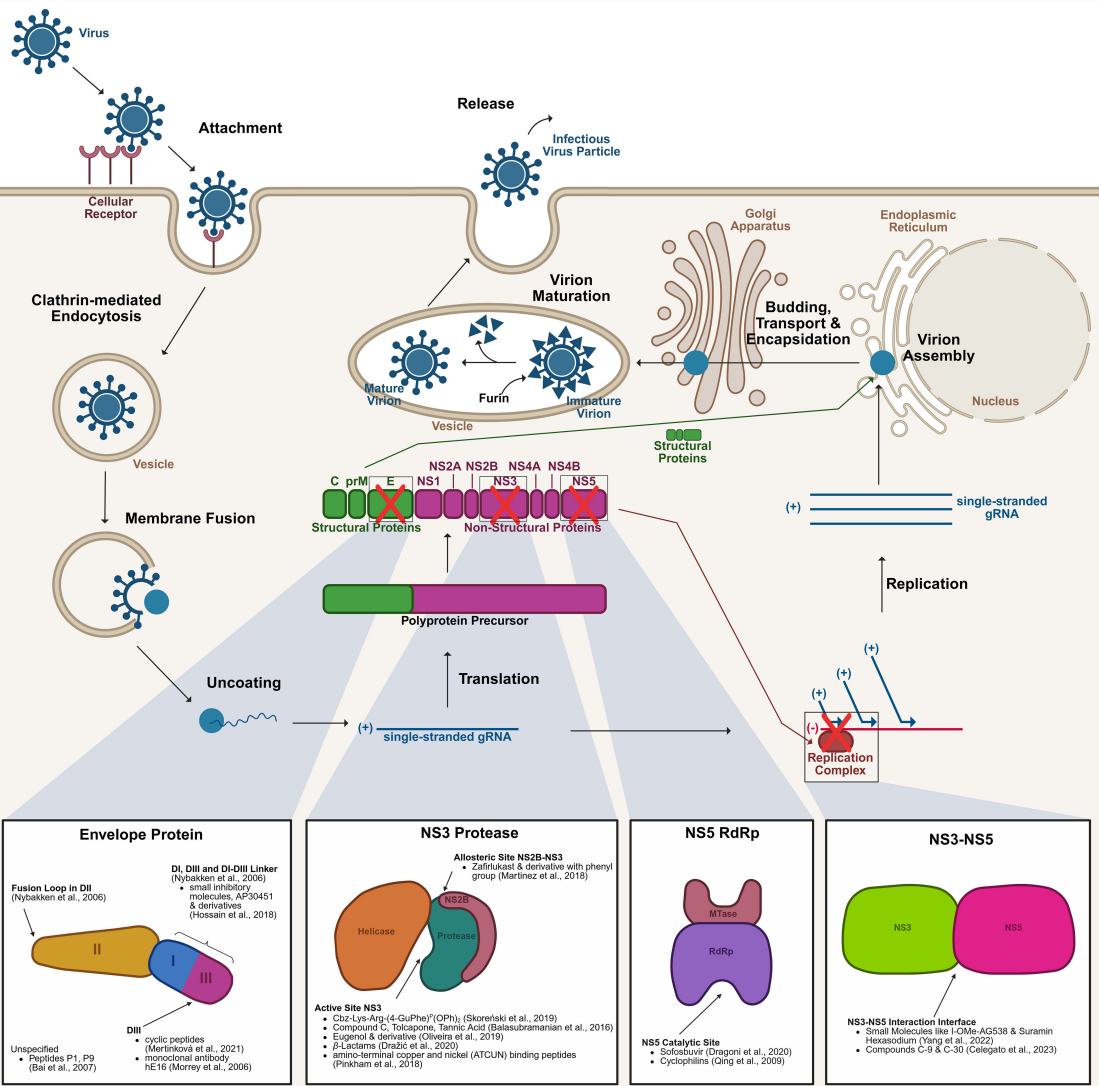
Life cycle of the West Nile virus and key drug targets. The WNV initiates infection by binding to an as-yet unidentified receptor on the host cell surface. Entry into the host cell is mediated via clathrin-dependent endocytosis. Following internalisation, the virus fuses with the vesicular membrane, releasing the gRNA into the cytoplasm. The gRNA is translated into a polyprotein precursor, which undergoes proteolytic cleavage to produce structural proteins (C, prM, E) and non-structural proteins (NS). The non-structural proteins form the replication complex, facilitating the synthesis of new gRNA. Structural proteins and replicated gRNA are assembled into nascent virions within the ER. The partially assembled virions are encapsidated and transported through the ERGIC to the Golgi apparatus. Final virion maturation occurs in the vesicles that are transported to the cell membrane, where furin cleaves prM to M, before the release of the newly formed infectious virus particles, which can now infect additional host cells. Several therapeutic targets have been identified along this pathway, such as the E protein, NS3 protease, NS5 RdRp, and the NS3-NS5 interaction interface. C, Capsid protein; E, Envelope protein; ER, Endoplasmic Reticulum; ERGIC, ER-Golgi intermediate compartment; gRNA, genomic RNA; NS, Non-Structural protein; prM, pre-Membrane protein; RdRp, RNA-dependent RNA polymerase; WNV, West Nile Virus. The figure was created with the help of BioRender.com.

West Nile virus (WNV) belongs to the genus *Flavivirus* (Lindenbach et al., 2007; Dong et al., 2008). It is a spherical, enveloped virus (~50 nm diameter) with a single-stranded, positive-sense RNA genome (~11 kb) encoding structural (Capsid, C; Envelope, E; pre-Membrane, prM) and non-structural (NS1 – NS5) proteins critical for replication, assembly, and immune evasion (Colpitts et al., 2012) (**Figure 1**). The structural proteins facilitate viral encapsulation and entry. The E protein mediates receptor binding and membrane fusion, while the prM protein stabilises immature virions, later cleaved into the membrane (M) protein for infectivity (Saiz et al., 2021). Non-structural proteins are vital for replication and immune modulation. NS1 assist replication complex formation and interferes with Toll-like receptor 3 (TLR3) signalling, NS2A/NS4A reshape host membranes for RNA replication and suppress interferon responses (Liu et al., 2005; Wilson et al., 2008; Londono-Renteria and Colpitts, 2016; Saiz et al., 2021). The multifunctional NS3 protease and helicase processes viral polyprotein and unwinds RNA, while NS5, the RNA-dependent RNA polymerase (RdRp), ensures genome replication and RNA capping. NS5 is the most conserved protein among flaviviruses, making it a key therapeutic target (Londono-Renteria and Colpitts, 2016; Habarugira et al., 2020; Saiz et al., 2021). WNV infects host cells via clathrin-mediated endocytosis. Endosomal membrane fusion releases the nucleocapsid into the cytoplasm, where RNA replication occurs at vesicle packets formed by rearranged endoplasmic reticulum (ER) membranes (Krishnan et al., 2007; van der Schaar et al., 2008; Londono-Renteria and Colpitts, 2016; Saiz et al., 2021). Immature virions assemble and mature through host protease cleavage before being released by exocytosis (Brinton, 2013; Londono-Renteria and Colpitts, 2016; Martin and Nisole, 2020; Saiz et al., 2021). Transmission primarily involves mosquitoes that acquire the virus from infected birds, its primary reservoirs, and spread it to humans and other mammals, which are dead-end hosts due to insufficient viremia (Colpitts et al., 2012; Ahlers and Goodman, 2018). Migratory birds likely drive WNV dissemination by interacting with resident bird populations, sustaining mosquito vectors, and maintaining zoonotic risks (Cendejas and Goodman, 2024; Lima et al., 2024).

This review examines recent advances in drug development targeting WNV’s E protein, which blocks viral entry, and NS3 and NS5, essential replication proteins with conserved sites that enable precise inhibitor design (Lescar et al., 2008; Fernandes et al., 2021), making them key targets for antiviral therapy.

## Drugs - envelope protein

The E protein, essential for virion assembly and host cell entry, is a critical drug target. Its DIII domain, responsible for receptor binding, has been targeted by entry inhibitors (**Figure 1**) (Nybakken et al., 2006; Saiz et al., 2021). Mertinková et al. (Mertinková et al., 2021) identified four cyclic peptides (CTKTDVHFC, CIHSSTRAC, CTYENHRTC, CLAQSHPLC) with affinity for DIII using *in silico* approach. These peptides exhibited neutralising activity *in vitro* with no detectable toxicity or haemolytic effects (Mertinková et al., 2021). Similarly, Bai et al. (Bai et al., 2007). demonstrated 50% of WNV infection inhibition by Peptide P1 (DTRACDVIALLCHLNT) at 67 µM *in vitro*, while its derivative P9 (CDVIALLACHLNT) achieved 50% inhibition at 2.6 µM. P9 also reduced viremia and brain viral loads in mice by crossing the blood-brain barrier, enhancing survival rates in lethal WNV challenges. Morrey et al. (Morrey et al., 2006). investigated a humanised monoclonal antibody (mAb; hE16) targeting DIII in a hamster model. A single intraperitoneal dose of hE16 five days post-infection achieved 80% survival rates, increasing to 88% when administered directly into the brain. Even hamsters with high WNV levels in cerebrospinal fluid were protected when treated. This suggests mAbs can neutralise WNV in the brain, likely facilitated by BBB alterations during encephalitis (Morrey et al., 2006).

There are also other regions in the E protein that are worth to be included in antiviral studies. Nybakken et al. (Nybakken et al., 2006). emphasise the significance of the DI-DIII linker, a region vital for both the fusion transition and the maturation process of viral particles, making it a promising candidate for further investigation. Additionally, the fusion loop in DII is another key target; it facilitates the homodimerization of the WNV E protein within the mature virion, contributing to the protein’s structural stability and functional integrity (Nybakken et al., 2006). Building on earlier work by Noueiry et al. (Noueiry et al., 2007), Hossain et al. (Hossain et al., 2018) highlight the potential of targeting specific regions within the E protein using small inhibitory molecules, such as AP30451 and its derivatives (**Figure 1**). These compounds have been shown to specifically inhibit flavivirus RNA translation and block the WNV replicon in ELISA *in vitro* assays in multiple cell lines. It was shown that these compounds bind to the DIII and DI domains of the E protein as well as regions proximal to the DI-DIII linker. Their findings underline the importance of these structural elements not only for viral function but also as promising targets for antiviral drug development.

While monoclonal antibodies like hE16 offer high specificity and potent neutralisation, their use can be limited by high production costs and delivery challenges, particularly for brain penetration. In contrast, small molecules can be more cost-effective, orally bioavailable, and capable of targeting intracellular processes, though they may face challenges related to resistance and off-target effects. These trade-offs highlight the need for a balanced approach in antiviral drug development (Wan, 2016; Desai et al., 2023; Wu et al., 2024). Though the E protein remains a critical entry target, non-structural proteins offer additional avenues for antiviral therapies by disrupting viral replication.

## Drugs – non-structural proteins

The NS3 protease plays a critical role in viral replication through its protease and helicase functions, and forms a serine protease complex with the cofactor NS2B, enabling the cleavage of non-structural proteins from the viral polyprotein, a crucial step for viral particle assembly (Hammamy et al., 2013; Lim and Shi, 2013; Bhakat et al., 2014) (**Figure 1**). This proteolytic activity is vital to produce functional viral proteins, facilitating effective viral replication and contributing to the overall pathogenicity of the virus. Consequently, in recent years, various experimental approaches have been explored to inhibit the NS3 functions, highlighting its potential as a target for antiviral drug development.

The FDA-approved asthma treatment molecule, zafirlukast, and its derivatives were evaluated for their potential to inhibit the NS3 protease (Martinez et al., 2018). Zafirlukast, composed of a toluic acid-*N*-aryl sulfonamide and a cyclopentyl carbamate linked by an indole backbone, binds to an NS3 allosteric site via the sulphonamide group and the aromatic ring inhibiting its protease activity. Zafirlukast occupation of allosteric site also hinders the docking of NS2B to NS3 inhibiting its function. Furthermore, *in vitro* studies demonstrated even better inhibition when the cyclopentyl group was replaced with a phenyl group. These findings were supported by both *in vitro* and *in silico* approaches, highlighting the critical structural components of zafirlukast that contribute to its inhibitory activity (Martinez et al., 2018).

The study by Skoreński et al. (Skoreński et al., 2019) screened various α-aminoalkylphosphonate diphenyl esters and their peptidyl derivatives for their ability to inhibit NS3 protease. Among the compounds evaluated, Cbz-Lys-Arg-(4-GuPhe)^P^(OPh)_2_ emerged as the most potent inhibitor. This molecule specifically targets the serine active site of the NS3 protease, leading to irreversible inhibition by forming a slowly hydrolysing protease-inhibitor complex (Skoreński et al., 2019) (**Figure 1**).

Balasubramanian et al. (Balasubramanian et al., 2016) further explored the NS3 protease as a drug target using an *in vitro* protease assay. They screened tolcapone, tannic acid, and compound C (a catechol derivative). In high-throughput screening (HTS), all three compounds exhibited strong inhibition of WNV protease activity, with C, F (tolcapone), and G (tannic acid) showing 98%, 99%, and 98% inhibition, respectively, at 10 µM concentrations. These inhibitors acted competitively, binding to the active site of the protease. *In silico* models using the DENV protease revealed that tannic acid had the highest binding efficiency, followed by compound C and tolcapone. While tannic acid, a phytochemical with historical use in natural medicine, has demonstrated broad antiviral properties, including protection against dengue virus, tolcapone’s application is limited due to its known hepatotoxicity, despite its effectiveness as a protease inhibitor (Balasubramanian et al., 2016) (**Figure 1**).

A eugenol derivative, namely 4-(3-(4-allyl-2-methoxyphenoxy)-propyl)-1-(2-bromobenzyl)-1H-1,2,3- triazole, was identified as a promising lead compound for inhibiting the NS3 protease. Eugenol, widely known for its anti-inflammatory, antiseptic, anaesthetic, and analgesic effects at low concentrations, is commonly used in dental products such as toothpastes and mouthwashes. The modification of eugenol has shown potent antiviral activity with low cytotoxicity *in vitro*. Molecular docking studies revealed that the eugenol derivative effectively binds to the catalytic triad of the NS3 protease. Given its natural origin, favourable safety profile, and specific recognition pattern in the protease, the eugenol derivative holds potential as a promising antiviral agent against *Flavivirus* infections, including West Nile virus (de Oliveira et al., 2019).

In the study of Dražić et al (Dražić et al., 2020), various peptide-*β*-lactams were used to inhibit the NS3 protease of WNV. The findings show that tripeptide-bound *β*-lactams, which previously showed affinity towards flavivirus NS3 proteases, are capable of inhibition by binding the active site. Tripeptide-bound *β*-lactams exhibit two distinct mechanisms of action. In the first mechanism, it forms a covalent and reversible bond with the catalytic serine of the NS3 protease. Upon release, the β-lactam ring undergoes opening. In the second mechanism, the amide bonds between lysine and benzyloxyphenylglycine are cleaved before release (Dražić et al., 2020) (**Figure 1**).

The strategy, which is used by Pinkham et al. (Pinkham et al., 2018), aims at the modification of the catalytic site of NS3 protease. Attenuation of the protease was achieved through use of different metallopeptides with benzoylated and naphtoylated N-terminal capping by irreversible oxidation of amino acid residues of the protease which are essential for substrate binding and catalysis. The study utilized amino-terminal copper and nickel (ATCUN) binding peptides, which have already been described as catalytically active protease inhibitors (Joyner and Cowan, 2011; Joyner et al., 2012; Joyner et al., 2013; Pinkham et al., 2018). The ATCUN motif was introduced by modifying the C-terminus of the WNV protease binding domain, where the N-terminal glycine of the tripeptide Gly-Gly-His was replaced with D-2,3-diaminopropionic acid (DDap), resulting in the sequence DDap-Gly-His. This targeting sequence was coupled through the 3’ amine of DDap, allowing the 2’ amine to coordinate with copper ions. Additionally, DDap-Gly-His was attached to targeting domains using polyglycine linkers of various lengths, optimizing the interaction and efficacy of the metallopeptides against the NS3 protease. Both, benzoylated and naphtoylated metallopeptides, are capable of oxidation of amino acid residues in the binding pocket important for catalysis and residues important for substrate binding, attenuating the NS3 protease in its function in viral replication (Pinkham et al., 2018).

NS5, with its dual methyltransferase (MTase) and RNA-dependent RNA polymerase (RdRp) functions, plays a pivotal role in the virus’ life cycle (Koonin, 1993; Tan et al., 1996; Guyatt et al., 2001; Egloff et al., 2002; Ray et al., 2006; Malet et al., 2007; Zhou et al., 2007; Davidson, 2009; Lim and Shi, 2013; Bhakat et al., 2014). Recent studies have considered on identifying compounds that inhibit NS5’s enzymatic activities, with various experimental approaches being explored to enhance the efficacy of antiviral therapies targeting this critical protein. Sofosbuvir is a licensed nucleotide analogue targeting the RdRp of hepatitis C virus (HCV), and has already shown successful inhibition *in vitro* and *in vivo* towards DENV and ZIKV (Lam et al., 2012; Sacramento et al., 2017; Xu et al., 2017; Mesci et al., 2018). It is proposed that sofosbuvir binds to the catalytic site of WNV RdRp by forming a wobble-like hydrogen bond with the uracil base of the RNA template (Dragoni et al., 2020), inhibiting the enzyme from its activity. Although this drug might be promising against WNV, the viral RdRp shows escape pathways against sofosbuvir through specific mutations in the active site of the enzyme (Dragoni et al., 2020) (**Figure 1**).

Adding to the therapeutic exploration of NS5, Qing et al. (Qing et al., 2009) highlights the role of cyclophilins (CyPs), a family of peptidyl-prolyl isomerases (PPIases), in viral replication. CyPs catalyse the isomerisation of peptide bonds at proline residues, facilitating proper protein folding. CyP interacts directly with the WNV genomic RNA and NS5 within the replication complex, acting as a molecular chaperone that maintains the active conformation of the complex to enhance viral RNA synthesis. The study demonstrates that cyclosporine (Cs), an 11-amino-acid cyclic peptide, inhibits the PPIase activity of CyP, thereby blocking its interaction with NS5 affecting the step of viral RNA synthesis. Knockdown experiments and viral RNA measurements further establish the importance of CyP in the replication process. While CyPs have been implicated in the replication of other viruses such as HIV and HCV, their involvement in the WNV lifecycle reinforces their significance as a host-derived target for therapeutic interventions. However, as the therapeutic dose of Cs remains unestablished, and *in vivo* studies are lacking, its clinical potential against WNV is yet to be determined, limiting current findings to *in vitro* assays (Qing et al., 2009).

The NS3-NS5 interaction is crucial for viral replication, making it a key target for antiviral strategies. Disrupting this interface with small molecules or peptides could block replication complex assembly, reducing viral replication and pathogenicity. Drug discovery efforts focus on identifying inhibitors to target this interaction has been done (Kapoor et al., 1995; Chen et al., 1997a; Johansson et al., 2001; Yon et al., 2005; Takahashi et al., 2012; Tay et al., 2015; Brand et al., 2017; Yang et al., 2022). Yang et al. (Yang et al., 2022) demonstrates that I-OMe tyrphostin AG538 (I-OMe-AG538) and suramin hexasodium (SHS), can inhibit the interaction of NS3 with NS5 of WNV *in vitro*. Due to specific side effects observed in humans, SHS has not received FDA approval. Nevertheless, it is a known anti-viral drug, blocking the replication of various viruses (Chen et al., 1997b; Ren et al., 2014; Albulescu et al., 2015; Ho et al., 2015; Henß et al., 2016; Albulescu et al., 2017; Salgado-Benvindo et al., 2020). It is suggested that both small molecules bind directly to NS5, destabilizing its structure and preventing its interaction with NS3, thereby inhibiting viral replication (Yang et al., 2022). Celegato et al. (Celegato et al., 2023) identified hit compounds C-9 (ZINC1333392 in ZINK15 docking database; C23H27N3O6S2) and C-30 (ZINC19598270; C18H11F5N4O), which inhibited DENV replication *in vitro* without cytotoxicity using digital docking. Compounds C-9, C-24 (ZINC56798609; C14H20N4O3), and C-30 also inhibited WNV replication in cells, demonstrating broad-spectrum antiviral potential. In mouse models, C-30 significantly reduced viral loads in the spleen and brain, effectively decreasing DENV replication in key tissues, including the brain. These compounds disrupt the NS3-NS5 interaction in a concentration-dependent manner. Their broad-spectrum activity suggests they may also be effective against WNV *in vivo*. This underscores the promise of targeting protein-protein interactions in flavivirus drug development (Celegato et al., 2023) (**Figure 1**).

The diverse range of antiviral strategies targeting WNV highlights the complexity of disrupting viral replication. While individual inhibitors show promise, mostly *in vitro* so far, their efficacy could be enhanced by combining multiple drugs that target different viral proteins, such as NS3 and NS5, to prevent escape mutations and improve treatment outcomes. Future studies should also explore such combination therapies to develop more robust antiviral approaches against WNV.

## Vaccines

As of now, no WNV vaccine has progressed beyond phase I or II clinical trials for humans, with candidates often requiring multiple doses and boosters (Principi and Esposito, 2024). ChimeriVax-WN02 (Sanofi Pasteur; NCT00442169, NCT00746798), a live attenuated chimeric vaccine, demonstrated seroconversion rates above 90% after a single dose in Phase II clinical trials (Dayan et al., 2013; Gould et al., 2023; Principi and Esposito, 2024). Several phase I clinical trials have evaluated alternative vaccine approaches, each with distinct immunogenic profiles. WN/DEN4-3’Δ30, a live attenuated chimeric vaccine, demonstrated seroconversion rates ranging from 55% to 95% depending on the dosing schedule in different clinical trials (NCT00094718, NCT00537147, NCT02186626) (Durbin et al., 2013; Gould et al., 2023; Principi and Esposito, 2024). DNA-based candidates, such as VRC-WNVDNA017-00-VP (NCT00106769) and VRC-WNVDNA020-00-VP (NCT00300417), have demonstrated strong neutralising antibody responses, with seroconversion rates exceeding 96% after a three-dose regimen (Gould et al., 2023; Principi and Esposito, 2024). Notable advancements include recombinant subunit vaccines, such as one using the truncated E protein (rWNV-80E) combined with adjuvants, which elicited strong humoral and cellular immunity in mice (Du et al., 2023; Gould et al., 2023; Principi and Esposito, 2024). Inactivated whole-virus formulations, including HydroVax-001 (NCT02337868), have shown moderate seroconversion rates (31–50%) following two doses, while a formalin-inactivated vaccine induced peak antibody responses after a booster dose (Gould et al., 2023; Principi and Esposito, 2024). Despite promising progress, no candidate has advanced beyond early trials, highlighting challenges in immunogenicity, dosing, and scalability for human use. While WNV exhibit less genetic variability when compared to dengue virus (DENV), its unpredictable infection course makes it difficult to assess vaccine efficacy, hindering clinical trial progression (Gould et al., 2023). Notably, none of these vaccine candidates have been reported to have adverse effects and are generally described as safe.

Both WNV and dengue virus belong to the *Flavivirus* genus, sharing similar structural and immunological features (Brand et al., 2017; Principi and Esposito, 2024). While no WNV vaccine has been approved for humans, two dengue vaccines, Dengvaxia^®^ and Qdenga^®^, have been licensed, providing valuable insights for WNV vaccine development. Dengvaxia^®^ is a live recombinant tetravalent vaccine, offering 75% protection against DENV-3 but only 34% for DENV-2, while Qdenga^®^ is a live attenuated version containing all serotypes which reaches 98% for DENV-2 (Principi and Esposito, 2024; World Health Organization, 2024; Zoetis, 2024). This suggests that similar strategies could help address WNV vaccine challenges in immunogenicity and scalability. The progression of ChimeriVax-WN02 to phase II trials marks a significant step forward despite ongoing hurdles.

In contrast, four vaccines have been approved for horses, including inactivated virus and canarypox-vectored platforms, demonstrating success in veterinary medicine (West Nile-Innovator^®^ by Zoetis, US; RECOMBITEK^®^ by MERIAL Ltd., US) (Merial Ltd, 2023; Zoetis, 2024). West Nile-Innovator^®^ showed 94% protection against viremia, while RECOMBITEK^®^ induced cell-mediated immunity and neutralizing antibodies (Ng et al., 2003; El Garch et al., 2008; Rossi et al., 2010; Filette et al., 2012; Pinto et al., 2013; Merial Ltd., 2023; Merck Animal Health, 2024; Zoetis, 2024).

## Conclusion

This review highlights significant advancements in developing antiviral drugs against WNV while emphasizing the need for further therapeutic development. Despite progress in computational modelling and *in vitro* studies targeting WNV’s proteins, *in vivo* assays remain limited, highlighting a gap in experimental validation compared to flaviviruses like YFV, DENV, and ZIKV. With WNV spreading to northern regions due to climate change, more *in vivo* experimentation and validated animal models are urgently needed to substantiate findings and expedite the development of effective antivirals and vaccines. Such efforts could not only address the growing WNV threat but also contribute to therapeutics for other flaviviruses, given their conserved proteins.

## References

[B1] Ahlers L. R. H. Goodman A. G. (2018). The immune responses of the animal hosts of west nile virus: A comparison of insects, birds, and mammals. Front. Cell Infect. Microbiol 8. doi: 10.3389/fcimb.2018.00096 PMC5891621 29666784

[B2] Albulescu I. C. Kovacikova K. Tas A. Snijder E. J. van Hemert M. J. (2017). Suramin inhibits Zika virus replication by interfering with virus attachment and release of infectious particles. Antiviral Res. 143, 230–236. doi: 10.1016/j.antiviral.2017.04.016 28461070

[B3] Albulescu I. C. van Hoolwerff M. Wolters L. A. Bottaro E. Nastruzzi C. Yang S. C.. (2015). Suramin inhibits chikungunya virus replication through multiple mechanisms. Antiviral Res. 121, 39–46. doi: 10.1016/j.antiviral.2015.06.013 26112648

[B4] Bai F. Town T. Pradhan D. Cox J. Ashish Ledizet M.. (2007). Antiviral peptides targeting the west nile virus envelope protein. J. Virol 81, 2047–2055. doi: 10.1128/JVI.01840-06 17151121 PMC1797586

[B5] Bakonyi T. Haussig J. M. (2020). West Nile virus keeps on moving up in Europe. Eurosurveillance 25, 2001938. doi: 10.2807/1560-7917.ES.2020.25.46.2001938 33213684 PMC7678036

[B6] Balasubramanian A. Manzano M. Teramoto T. Pilankatta R. Padmanabhan R. (2016). High-throughput screening for the identification of small-molecule inhibitors of the flaviviral protease. Antiviral Res. 134, 6–16. doi: 10.1016/j.antiviral.2016.08.014 27539384 PMC5065773

[B7] Bhakat S. Karubiu W. Jayaprakash V. Soliman M. E. S. (2014). A perspective on targeting non-structural proteins to combat neglected tropical diseases: Dengue, West Nile and Chikungunya viruses. Eur. J. Med. Chem. 87, 677–702. doi: 10.1016/j.ejmech.2014.10.010 25305334

[B8] Brand C. Bisaillon M. Geiss B. J. (2017). Organization of the Flavivirus RNA replicase complex. WIREs RNA 8, e1437. doi: 10.1002/wrna.1437 PMC5675032 28815931

[B9] Brinton M. A. (2013). Replication cycle and molecular biology of the West Nile virus. Viruses 6, 13–53. doi: 10.3390/v6010013 24378320 PMC3917430

[B10] Celegato M. Sturlese M. Vasconcelos Costa V. Trevisan M. Lallo Dias A. S. Souza Passos I. B.. (2023). Small-molecule inhibitor of flaviviral NS3-NS5 interaction with broad-spectrum activity and efficacy *in vivo* . mBio 14, e0309722. doi: 10.1128/mbio.03097-22 36622141 PMC9973282

[B11] Cendejas P. M. Goodman A. G. (2024). Vaccination and control methods of west nile virus infection in equids and humans. Vaccines (Basel) 12, 485. doi: 10.3390/vaccines12050485 38793736 PMC11125624

[B12] Chen C. J. Kuo M. D. Chien L. J. Hsu S. L. Wang Y. M. Lin J. H. (1997a). RNA-protein interactions: involvement of NS3, NS5, and 3’ noncoding regions of Japanese encephalitis virus genomic RNA. J. Virol 71, 3466–3473. doi: 10.1128/JVI.71.5.3466-3473.1997 9094618 PMC191493

[B13] Chen Y. Maguire T. Hileman R. E. Fromm J. R. Esko J. D. Linhardt R. J.. (1997b). Dengue virus infectivity depends on envelope protein binding to target cell heparan sulfate. Nat. Med. 3, 866–871. doi: 10.1038/nm0897-866 9256277

[B14] Chowdhury P. Khan S. A. (2021). Global emergence of West Nile virus: Threat & preparedness in special perspective to India. Indian J. Med. Res. 154, 36–50. doi: 10.4103/ijmr.IJMR_642_19 34782529 PMC8715705

[B15] Colpitts T. M. Conway M. J. Montgomery R. R. Fikrig E. (2012). West Nile Virus: biology, transmission, and human infection. Clin. Microbiol Rev. 25, 635–648. doi: 10.1128/CMR.00045-12 23034323 PMC3485754

[B16] Davidson A. D. (2009). Chapter 2. New insights into flavivirus nonstructural protein 5. Adv. Virus Res. 74, 41–101. doi: 10.1016/S0065-3527(09)74002-3 19698895

[B17] Dayan G. H. Pugachev K. Bevilacqua J. Lang J. Monath T. P. (2013). Preclinical and clinical development of a YFV 17 D-based chimeric vaccine against West Nile virus. Viruses 5, 3048–3070. doi: 10.3390/v5123048 24351795 PMC3967160

[B18] DeFelice N. B. Little E. Campbell S. R. Shaman J. (2017). Ensemble forecast of human West Nile virus cases and mosquito infection rates. Nat. Commun. 8, 14592. doi: 10.1038/ncomms14592 28233783 PMC5333106

[B19] de Oliveira A. S. Gazolla P. A. R. Da Oliveira A. F. C. S. Pereira W. L. de S. Viol L. C. Da Maia A. F. S.. (2019). Discovery of novel West Nile Virus protease inhibitor based on isobenzonafuranone and triazolic derivatives of eugenol and indan-1,3-dione scaffolds. PloS One 14, e0223017. doi: 10.1371/journal.pone.0223017 31557229 PMC6762200

[B20] Desai M. Kundu A. Hageman M. Lou H. Boisvert D. (2023). Monoclonal antibody and protein therapeutic formulations for subcutaneous delivery: high-concentration, low-volume vs. low-concentration, high-volume. MAbs 15, 2285277. doi: 10.1080/19420862.2023.2285277 38013454 PMC10793682

[B21] Dong H. Zhang B. Shi P.-Y. (2008). Terminal structures of West Nile virus genomic RNA and their interactions with viral NS5 protein. Virology 381, 123–135. doi: 10.1016/j.virol.2008.07.040 18799181

[B22] Dragoni F. Boccuto A. Picarazzi F. Giannini A. Giammarino F. Saladini F.. (2020). Evaluation of sofosbuvir activity and resistance profile against West Nile virus *in vitro* . Antiviral Res. 175, 104708. doi: 10.1016/j.antiviral.2020.104708 31931104

[B23] Dražić T. Kopf S. Corridan J. Leuthold M. M. Bertoša B. Klein C. D. (2020). Peptide-β-lactam inhibitors of dengue and west nile virus NS2B-NS3 protease display two distinct binding modes. J. Med. Chem. 63, 140–156. doi: 10.1021/acs.jmedchem.9b00759 31804823

[B24] Du Y. Deng Y. Zhan Y. Yang R. Ren J. Wang W.. (2023). The recombinant truncated envelope protein of West Nile virus adjuvanted with Alum/CpG induces potent humoral and T cell immunity in mice. Biosafety Health 5, 300–307. doi: 10.1016/j.bsheal.2023.06.003 40078908 PMC11894981

[B25] Durbin A. P. Wright P. F. Cox A. Kagucia W. Elwood D. Henderson S.. (2013). The live attenuated chimeric vaccine rWN/DEN4Δ30 is well-tolerated and immunogenic in healthy flavivirus-naïve adult volunteers. Vaccine 31, 5772–5777. doi: 10.1016/j.vaccine.2013.07.064 23968769 PMC3833717

[B26] ECDC (2024a). Surveillance atlas of infectious diseases. Available online at: https://atlas.ecdc.europa.eu/public/index.aspx (Accessed October 2, 2024).

[B27] ECDC (2024b). West Nile virus infections in humans, 2012-2022.

[B28] Egloff M.-P. Benarroch D. Selisko B. Romette J.-L. Canard B. (2002). An RNA cap (nucleoside-2’-O-)-methyltransferase in the flavivirus RNA polymerase NS5: crystal structure and functional characterization. EMBO J. 21, 2757–2768. doi: 10.1093/emboj/21.11.2757 12032088 PMC125380

[B29] El Garch H. Minke J. M. Rehder J. Richard S. Edlund Toulemonde C. Dinic S.. (2008). A West Nile virus (WNV) recombinant canarypox virus vaccine elicits WNV-specific neutralizing antibodies and cell-mediated immune responses in the horse. Vet Immunol Immunopathol 123, 230–239. doi: 10.1016/j.vetimm.2008.02.002 18372050

[B30] Erazo D. Grant L. Ghisbain G. Marini G. Colón-González F. J. Wint W.. (2024). Contribution of climate change to the spatial expansion of West Nile virus in Europe. Nat. Commun. 15, 1196. doi: 10.1038/s41467-024-45290-3 38331945 PMC10853512

[B31] Farooq Z. Sjödin H. Semenza J. C. Tozan Y. Sewe M. O. Wallin J.. (2023). European projections of West Nile virus transmission under climate change scenarios. One Health 16, 100509. doi: 10.1016/j.onehlt.2023.100509 37363233 PMC10288058

[B32] Fernandes P. O. Chagas M. A. Rocha W. R. Moraes A. H. (2021). Non-structural protein 5 (NS5) as a target for antiviral development against established and emergent flaviviruses. Curr. Opin. Virol 50, 30–39. doi: 10.1016/j.coviro.2021.07.001 34340199

[B33] Filette M. de Ulbert S. Diamond M. Sanders N. N. (2012). Recent progress in West Nile virus diagnosis and vaccination. Vet Res 43, 16. doi: 10.1186/1297-9716-43-16 22380523 PMC3311072

[B34] Giesen C. Herrador Z. Fernandez-Martinez B. Figuerola J. Gangoso L. Vazquez A.. (2023). A systematic review of environmental factors related to WNV circulation in European and Mediterranean countries. One Health 16, 100478. doi: 10.1016/j.onehlt.2022.100478 37363246 PMC10288031

[B35] Gould C. V. Staples J. E. Huang C. Y.-H. Brault A. C. Nett R. J. (2023). Combating west nile virus disease — Time to revisit vaccination. N Engl. J. Med. 388, 1633–1636. doi: 10.1056/NEJMp2301816 37125778 PMC11627013

[B36] Guyatt K. J. Westaway E. G. Khromykh A. A. (2001). Expression and purification of enzymatically active recombinant RNA-dependent RNA polymerase (NS5) of the flavivirus Kunjin. J. Virol Methods 92, 37–44. doi: 10.1016/s0166-0934(00)00270-6 11164916

[B37] Habarugira G. Suen W. W. Hobson-Peters J. Hall R. A. Bielefeldt-Ohmann H. (2020). West nile virus: an update on pathobiology, epidemiology, diagnostics, control and “One health” Implications. Pathogens 9. doi: 10.3390/pathogens9070589 PMC7400489 32707644

[B38] Hammamy M. Z. Haase C. Hammami M. Hilgenfeld R. Steinmetzer T. (2013). Development and characterization of new peptidomimetic inhibitors of the West Nile virus NS2B-NS3 protease. ChemMedChem 8, 231–241. doi: 10.1002/cmdc.201200497 23307694

[B39] Henß L. Beck S. Weidner T. Biedenkopf N. Sliva K. Weber C.. (2016). Suramin is a potent inhibitor of Chikungunya and Ebola virus cell entry. Virol J. 13, 149. doi: 10.1186/s12985-016-0607-2 27581733 PMC5007819

[B40] Ho Y.-J. Wang Y.-M. Lu J. Wu T.-Y. Lin L.-I. Kuo S.-C.. (2015). Suramin inhibits chikungunya virus entry and transmission. PloS One 10, e0133511. doi: 10.1371/journal.pone.0133511 26208101 PMC4514758

[B41] Hossain M. U. Keya C. A. Das K. C. Hashem A. Omar T. M. Khan M. A.. (2018). An immunopharmacoinformatics approach in development of vaccine and drug candidates for west nile virus. Front. Chem. 6. doi: 10.3389/fchem.2018.00246 PMC6043868 30035107

[B42] Johansson M. Brooks A. J. Jans D. A. Vasudevan S. G. (2001). A small region of the dengue virus-encoded RNA-dependent RNA polymerase, NS5, confers interaction with both the nuclear transport receptor importin-β and the viral helicase, NS3. J. Gen. Virol. 82, 735–745. doi: 10.1099/0022-1317-82-4-735 11257177

[B43] Joyner J. C. Cowan J. A. (2011). Targeted cleavage of HIV RRE RNA by Rev-coupled transition metal chelates. J. Am. Chem. Soc. 133, 9912–9922. doi: 10.1021/ja203057z 21585196 PMC3387528

[B44] Joyner J. C. Hocharoen L. Cowan J. A. (2012). Targeted catalytic inactivation of angiotensin converting enzyme by lisinopril-coupled transition-metal chelates. J. Am. Chem. Soc. 134, 3396–3410. doi: 10.1021/ja208791f 22200082 PMC3401419

[B45] Joyner J. C. Hodnick W. F. Cowan A. S. Tamuly D. Boyd R. Cowan J. A. (2013). Antimicrobial metallopeptides with broad nuclease and ribonuclease activity. Chem. Commun. (Camb) 49, 2118–2120. doi: 10.1039/c3cc38977d 23380915 PMC3632407

[B46] Kapoor M. Zhang L. Ramachandra M. Kusukawa J. Ebner K. E. Padmanabhan R. (1995). Association between NS3 and NS5 proteins of dengue virus type 2 in the putative RNA replicase is linked to differential phosphorylation of NS5 *. J. Biol. Chem. 270, 19100–19106. doi: 10.1074/jbc.270.32.19100 7642575

[B47] Koonin E. V. (1993). Computer-assisted identification of a putative methyltransferase domain in NS5 protein of flaviviruses and lambda 2 protein of reovirus. J. Gen. Virol 74, 733–740. doi: 10.1099/0022-1317-74-4-733 8385698

[B48] Kramer L. D. Ciota A. T. Kilpatrick A. M. (2019). Introduction, spread, and establishment of west nile virus in the americas. J. Med. Entomol 56, 1448–1455. doi: 10.1093/jme/tjz151 31549719 PMC7182919

[B49] Krishnan M. N. Sukumaran B. Pal U. Agaisse H. Murray J. L. Hodge T. W.. (2007). Rab 5 is required for the cellular entry of dengue and west nile viruses. J. Virol 81, 4881–4885. doi: 10.1128/jvi.02210-06 17301152 PMC1900185

[B50] Lam A. M. Espiritu C. Bansal S. Micolochick Steuer H. M. Niu C. Zennou V.. (2012). Genotype and subtype profiling of PSI-7977 as a nucleotide inhibitor of hepatitis C virus. Antimicrobial Agents Chemotherapy 56, 3359–3368. doi: 10.1128/aac.00054-12 22430955 PMC3370800

[B51] Lanciotti R. S. Ebel G. D. Deubel V. Kerst A. J. Murri S. Meyer R.. (2002). Complete genome sequences and phylogenetic analysis of west nile virus strains isolated from the United States, europe, and the middle east. Virology 298, 96–105. doi: 10.1006/viro.2002.1449 12093177

[B52] Lanciotti R. S. Roehrig J. T. Deubel V. Smith J. Parker M. Steele K.. (1999). Origin of the West Nile virus responsible for an outbreak of encephalitis in the northeastern United States. Science 286, 2333–2337. doi: 10.1126/science.286.5448.2333 10600742

[B53] Lescar J. Luo D. Xu T. Sampath A. Lim S. P. Canard B.. (2008). Towards the design of antiviral inhibitors against flaviviruses: the case for the multifunctional NS3 protein from Dengue virus as a target. Antiviral Res. 80, 94–101. doi: 10.1016/j.antiviral.2008.07.001 18674567

[B54] Lim S. P. Shi P.-Y. (2013). West Nile virus drug discovery. Viruses 5, 2977–3006. doi: 10.3390/v5122977 24300672 PMC3967157

[B55] Lima P. C. Lustosa R. Romano A. P. M. Araújo P. C. de Oliveira Passos P. H. Ramos D. G.. (2024). Serological evidence of West Nile virus infection in wild birds in the area of the first confirmed human case of West Nile fever in Brazil. Revista Pan-Amazônica de Saúde 15.

[B56] Lindenbach B. D. Thiel H. J. Rice C. M. (2007). “Flaviviridae: The virus and their replication,” in Fields virology. Eds. Knipe D. M. Howley P. M. (Lippincott-Raven, Philadelphia, PA), 1101.

[B57] Liu W. J. Wang X. J. Mokhonov V. V. Shi P.-Y. Randall R. Khromykh A. A. (2005). Inhibition of interferon signaling by the New York 99 strain and Kunjin subtype of West Nile virus involves blockage of STAT1 and STAT2 activation by nonstructural proteins. J. Virol 79, 1934–1942. doi: 10.1128/JVI.79.3.1934-1942.2005 15650219 PMC544092

[B58] Londono-Renteria B. Colpitts T. M. (2016). A brief review of west nile virus biology. Methods Mol. Biol. 1435, 1–13. doi: 10.1007/978-1-4939-3670-0_1 27188545

[B59] Lorenz C. Chiaravalloti-Neto F. (2022). Why are there no human West Nile virus outbreaks in South America? Lancet Reg. Health Am. 12, 100276. doi: 10.1016/j.lana.2022.100276 36776433 PMC9903813

[B60] Malet H. Egloff M.-P. Selisko B. Butcher R. E. Wright P. J. Roberts M.. (2007). Crystal structure of the RNA polymerase domain of the West Nile virus non-structural protein 5. J. Biol. Chem. 282, 10678–10689. doi: 10.1074/jbc.M607273200 17287213

[B61] Martin M.-F. Nisole S. (2020). West nile virus restriction in mosquito and human cells: A virus under confinement. Vaccines (Basel) 8. doi: 10.3390/vaccines8020256 PMC7350012 32485916

[B62] Martinez A. A. Espinosa B. A. Adamek R. N. Thomas B. A. Chau J. Gonzalez E.. (2018). Breathing new life into West Nile virus therapeutics; discovery and study of zafirlukast as an NS2B-NS3 protease inhibitor. Eur. J. Med. Chem. 157, 1202–1213. doi: 10.1016/j.ejmech.2018.08.077 30193218

[B63] May F. J. Davis C. T. Tesh R. B. Barrett A. D. T. (2011). Phylogeography of West Nile virus: from the cradle of evolution in Africa to Eurasia, Australia, and the Americas. J. Virol 85, 2964–2974. doi: 10.1128/JVI.01963-10 21159871 PMC3067944

[B64] Merial Ltd. (2023). RECOMBITEK^®^ Equine west nile virus (MERIAL LTD.). Available online at: https://datasheets.scbt.com/sc-359390_mfr.pdf (Accessed October 4, 2024).

[B65] Merck Animal Health. (2024). PRESTIGE® WNV. Available online at: https://www.merck-animal-health-usa.com/species/equine/products/prestige-wnv (Accessed October 04, 2024).

[B66] Mertinková P. Mochnáčová E. Bhide K. Kulkarni A. Tkáčová Z. Hruškovicová J.. (2021). Development of peptides targeting receptor binding site of the envelope glycoprotein to contain the West Nile virus infection. Sci. Rep. 11, 20131. doi: 10.1038/s41598-021-99696-w 34635758 PMC8505397

[B67] Mesci P. Macia A. Moore S. M. Shiryaev S. A. Pinto A. Huang C.-T.. (2018). Blocking Zika virus vertical transmission. Sci. Rep. 8, 1218. doi: 10.1038/s41598-018-19526-4 29352135 PMC5775359

[B68] Morrey J. D. Siddharthan V. Olsen A. L. Roper G. Y. Wang H. Baldwin T. J.. (2006). Humanized Monoclonal Antibody against West Nile Virus Envelope Protein Administered after Neuronal Infection Protects against Lethal Encephalitis in Hamsters. J. Infect. Dis. 194, 1300–1308. doi: 10.1086/508293 17041857

[B69] Ng T. Hathaway D. Jennings N. Champ D. Chiang Y. W. Chu H. J. (2003). Equine vaccine for West Nile virus. Dev Biol (Basel) 114, 221–227. 14677692

[B70] Noueiry A. O. Olivo P. D. Slomczynska U. Zhou Y. Buscher B. Geiss B.. (2007). Identification of novel small-molecule inhibitors of West Nile virus infection. J. Virol 81, 11992–12004. doi: 10.1128/JVI.01358-07 17715228 PMC2168801

[B71] Nybakken G. E. Nelson C. A. Chen B. R. Diamond M. S. Fremont D. H. (2006). Crystal structure of the West Nile virus envelope glycoprotein. J. Virol 80, 11467–11474. doi: 10.1128/JVI.01125-06 16987985 PMC1642602

[B72] Paz S. (2015). Climate change impacts on West Nile virus transmission in a global context. Philos. Trans. R Soc. Lond B Biol. Sci. 370. doi: 10.1098/rstb.2013.0561 PMC4342965 25688020

[B73] Pinkham A. M. Yu Z. Cowan J. A. (2018). Attenuation of west nile virus NS2B/NS3 protease by amino terminal copper and nickel binding (ATCUN) peptides. J. Med. Chem. 61, 980–988. doi: 10.1021/acs.jmedchem.7b01409 29301071

[B74] Pinto A. K. Richner J. M. Poore E. A. Patil P. P. Amanna I. J. Slifka M. K.. (2013). A hydrogen peroxide-inactivated virus vaccine elicits humoral and cellular immunity and protects against lethal West Nile virus infection in aged mice. J Virol 87, 1926–1936. doi: 10.1128/JVI.02903-12 23221549 PMC3571480

[B75] Principi N. Esposito S. (2024). Development of vaccines against emerging mosquito-vectored arbovirus infections. Vaccines (Basel) 12. doi: 10.3390/vaccines12010087 PMC10818606 38250900

[B76] Qing M. Yang F. Zhang B. Zou G. Robida J. M. Yuan Z.. (2009). Cyclosporine inhibits flavivirus replication through blocking the interaction between host cyclophilins and viral NS5 protein. Antimicrobial Agents Chemotherapy 53, 3226–3235. doi: 10.1128/AAC.00189-09 19451286 PMC2715601

[B77] Ray D. Shah A. Tilgner M. Guo Y. Zhao Y. Dong H.. (2006). West Nile virus 5’-cap structure is formed by sequential guanine N-7 and ribose 2’-O methylations by nonstructural protein 5. J. Virol 80, 8362–8370. doi: 10.1128/JVI.00814-06 16912287 PMC1563844

[B78] Ren P. Zou G. Bailly B. Xu S. Zeng M. Chen X.. (2014). The approved pediatric drug suramin identified as a clinical candidate for the treatment of EV71 infection-suramin inhibits EV71 infection *in vitro* and *in vivo* . Emerg Microbes Infect. 3, e62. doi: 10.1038/emi.2014.60 26038755 PMC4185360

[B79] Rossi S. L. Ross T. M. Evans J. D. (2010). West Nile virus. Clin Lab Med 30, 47–65. doi: 10.1016/j.cll.2009.10.006 20513541 PMC2905782

[B80] Sacramento C. Q. de Melo G. R. de Freitas C. S. Rocha N. Hoelz L. V. B. Miranda M.. (2017). The clinically approved antiviral drug sofosbuvir inhibits Zika virus replication. Sci. Rep. 7, 40920. doi: 10.1038/srep40920 28098253 PMC5241873

[B81] Saiz J.-C. Martín-Acebes M. A. Blázquez A. B. Escribano-Romero E. Poderoso T. Jiménez de Oya N. (2021). Pathogenicity and virulence of West Nile virus revisited eight decades after its first isolation. Virulence 12, 1145–1173. doi: 10.1080/21505594.2021.1908740 33843445 PMC8043182

[B82] Salgado-Benvindo C. Thaler M. Tas A. Ogando N. S. Bredenbeek P. J. Ninaber D. K.. (2020). Suramin inhibits SARS-coV-2 infection in cell culture by interfering with early steps of the replication cycle. Antimicrobial Agents Chemotherapy 64. doi: 10.1128/AAC.00900-20 PMC7526844 32513797

[B83] Semenza J. C. Rocklöv J. Ebi K. L. (2022). Climate change and cascading risks from infectious disease. Infect. Dis. Ther. 11, 1371–1390. doi: 10.1007/s40121-022-00647-3 35585385 PMC9334478

[B84] Sinigaglia A. Peta E. Riccetti S. Barzon L. (2020). New avenues for therapeutic discovery against West Nile virus. Expert Opin. Drug Discov. 15, 333–348. doi: 10.1080/17460441.2020.1714586 32017639

[B85] Skoreński M. Milewska A. Pyrć K. Sieńczyk M. Oleksyszyn J. (2019). Phosphonate inhibitors of West Nile virus NS2B/NS3 protease. J. Enzyme Inhib Med. Chem. 34, 8–14. doi: 10.1080/14756366.2018.1506772 30362835 PMC6211275

[B86] Takahashi H. Takahashi C. Moreland N. J. Chang Y.-T. Sawasaki T. Ryo A.. (2012). Establishment of a robust dengue virus NS3–NS5 binding assay for identification of protein–protein interaction inhibitors. Antiviral Res. 96, 305–314. doi: 10.1016/j.antiviral.2012.09.023 23072882

[B87] Tan B. H. Fu J. Sugrue R. J. Yap E. H. Chan Y. C. Tan Y. H. (1996). Recombinant dengue type 1 virus NS5 protein expressed in Escherichia coli exhibits RNA-dependent RNA polymerase activity. Virology 216, 317–325. doi: 10.1006/viro.1996.0067 8607261

[B88] Tay M. Y. Saw W. G. Zhao Y. Chan K. W. Singh D. Chong Y.. (2015). The C-terminal 50 amino acid residues of dengue NS3 protein are important for NS3-NS5 interaction and viral replication *. J. Biol. Chem. 290, 2379–2394. doi: 10.1074/jbc.M114.607341 25488659 PMC4303688

[B89] van der Schaar H. M. Rust M. J. Chen C. van der Ende-Metselaar H. Wilschut J. Zhuang X.. (2008). Dissecting the cell entry pathway of dengue virus by single-particle tracking in living cells. PloS Pathog. 4, e1000244. doi: 10.1371/journal.ppat.1000244 19096510 PMC2592694

[B90] Vogels C. B. Göertz G. P. Pijlman G. P. Koenraadt C. J. (2017). Vector competence of European mosquitoes for West Nile virus. Emerg Microbes Infect. 6, e96. doi: 10.1038/emi.2017.82 29116220 PMC5717085

[B91] Wan H. (2016). An overall comparison of small molecules and large biologics in ADME testing. ADMET DMPK 4, 1. doi: 10.5599/admet.4.1.276

[B92] Weaver S. C. (2013). Urbanization and geographic expansion of zoonotic arboviral diseases: mechanisms and potential strategies for prevention. Trends Microbiol 21, 360–363. doi: 10.1016/j.tim.2013.03.003 23910545 PMC5193003

[B93] Weaver S. C. Reisen W. K. (2010). Present and future arboviral threats. Antiviral Res. 85, 328–345. doi: 10.1016/j.antiviral.2009.10.008 19857523 PMC2815176

[B94] Wilson J. R. de Sessions P. F. Leon M. A. Scholle F. (2008). West Nile virus nonstructural protein 1 inhibits TLR3 signal transduction. J. Virol 82, 8262–8271. doi: 10.1128/JVI.00226-08 18562533 PMC2519649

[B95] World Health Organization (2024). Vaccines and immunization: Dengue. Available online at: https://www.who.int/news-room/questions-and-answers/item/dengue-vaccines (Accessed March 1, 2025).

[B96] Wu K. Kwon S. H. Zhou X. Fuller C. Wang X. Vadgama J.. (2024). Overcoming challenges in small-molecule drug bioavailability: A review of key factors and approaches. Int. J. Mol. Sci. 25. doi: 10.3390/ijms252313121 PMC11642056 39684832

[B97] Xu H.-T. Colby-Germinario S. P. Hassounah S. A. Fogarty C. Osman N. Palanisamy N.. (2017). Evaluation of Sofosbuvir (β-D-2′-deoxy-2′-α-fluoro-2′-β-C-methyluridine) as an inhibitor of Dengue virus replication. Sci. Rep. 7, 6345. doi: 10.1038/s41598-017-06612-2 28740124 PMC5524696

[B98] Yang S. N. Y. Maher B. Wang C. Wagstaff K. M. Fraser J. E. Jans D. A. (2022). High throughput screening targeting the dengue NS3-NS5 interface identifies antivirals against dengue, zika and west nile viruses. Cells 11. doi: 10.3390/cells11040730 PMC8870125 35203378

[B99] Yon C. Teramoto T. Mueller N. Phelan J. Ganesh V. K. Murthy K. H.. (2005). Modulation of the nucleoside triphosphatase/RNA helicase and 5′-RNA triphosphatase activities of dengue virus type 2 nonstructural protein 3 (NS3) by interaction with NS5, the RNA-dependent RNA polymerase *. J. Biol. Chem. 280, 27412–27419. doi: 10.1074/jbc.M501393200 15917225

[B100] Zhou Y. Ray D. Zhao Y. Dong H. Ren S. Li Z.. (2007). Structure and function of flavivirus NS5 methyltransferase. J. Virol 81, 3891–3903. doi: 10.1128/JVI.02704-06 17267492 PMC1866096

[B101] Zoetis (2024). WEST NILE-INNOVATOR^®^ - safety data sheet. Available online at: https://www.zoetisus.com/products/equine/west-nile-equine-vaccine-for-horses:~:text=WEST%20NILE%2DINNOVATOR%C2%AE%20vaccines%20contain%20the%20adjuvant%20MetaStim%C2%AE,immunity%20to%20West%20Nile%20virus (Accessed October 4, 2024).

